# Real-Time Underwater StereoFusion

**DOI:** 10.3390/s18113936

**Published:** 2018-11-14

**Authors:** Matija Rossi, Petar Trslić, Satja Sivčev, James Riordan, Daniel Toal, Gerard Dooly

**Affiliations:** 1 Centre for Robotics & Intelligent Systems, University of Limerick, Limerick V94 T9PX, Ireland; petar.trslic@ul.ie (P.T.); satja.sivcev@ul.ie (S.S.); daniel.toal@ul.ie (D.T.); gerard.dooly@ul.ie (G.D.); 2 School of Computing, Engineering, and Physical Sciences, University of the West of Scotland, Glasgow G72 0AG, UK; james.riordan@uws.ac.uk

**Keywords:** stereo, underwater, ROV, GPU, real-time, 3D, fusion, camera, tracking, vision

## Abstract

Many current and future applications of underwater robotics require real-time sensing and interpretation of the environment. As the vast majority of robots are equipped with cameras, computer vision is playing an increasingly important role it this field. This paper presents the implementation and experimental results of underwater StereoFusion, an algorithm for real-time 3D dense reconstruction and camera tracking. Unlike KinectFusion on which it is based, StereoFusion relies on a stereo camera as its main sensor. The algorithm uses the depth map obtained from the stereo camera to incrementally build a volumetric 3D model of the environment, while simultaneously using the model for camera tracking. It has been successfully tested both in a lake and in the ocean, using two different state-of-the-art underwater Remotely Operated Vehicles (ROVs). Ongoing work focuses on applying the same algorithm to acoustic sensors, and on the implementation of a vision based monocular system with the same capabilities.

## 1. Introduction

Applications of computer vision are rapidly growing across a wide spectrum of underwater operations. Vision systems are increasingly being used as the primary tool for inspection of underwater sites, in disciplines ranging from archaeology [1] and biology [2], to offshore engineering [3] and pipeline inspection [4]. This has been facilitated by the increasing industry adoption of remotely operated vehicles (ROV) and autonomous underwater vehicles (AUV) [5,6,7], which opens the door to many new applications for machine vision. A common task is robot navigation, for which underwater is challenging for many reasons, such as the lack of radio communications, including global navigation satellite systems (GNSS), and limited sensing technology compared to land or airborne vehicles. For this purpose, camera and acoustic sensor systems can be used to implement simultaneous localisation and mapping (SLAM) algorithms to complement inertial navigation systems (INS), which inevitably suffer from drift. If such algorithms prove sufficiently robust, vision systems may obviate the need for inertial navigation systems and replace them with image-based target referenced navigation [8].

An even more demanding task is robotic intervention, where work class ROVs equipped with underwater manipulators have traditionally been teleoperated from support vessels by human operators. Significant effort is currently being put towards the automation of such operations using computer vision [9,10,11,12]. In order for an intervention task to be carried out autonomously, it is necessary to know the structure of the scene around the target and the position of the robot relative to it. This makes it possible to then implement higher level features such as path planning, obstacle avoidance, and target identification. Additionally, even in the case of manual operations, providing an augmented feedback could increase the ROV pilot’s efficiency multiple times compared to a standard 2D camera stream, which is what is currently being used for teleoperation of manipulators. Due to offshore operations being particularly expensive, time consuming, and limited by other factors such as weather, making them more efficient is of great value [13].

The described scenarios would significantly benefit from, or even require, models of the underwater operating environment generated in real time [14]. Another common example is survey data which is usually post-processed, and is acquired without real-time feedback about its quality. This leads to situations where defects in the data, such as areas of interest not being fully covered, are discovered only after the survey.

This paper presents an underwater StereoFusion algorithm based on KinectFusion [15,16] as a reliable solution to the described requirements, capable of real-time dense 3D reconstruction and localisation using an underwater stereo camera. The main contributions of this work are the implementation of StereFusion, its application on both a custom-built and a commercial ROV, and its testing in fresh water and in the ocean, under very different visibility conditions.

Section 2 provides a brief background of previous work relevant to the development of the described system. Section 3 describes the algorithm itself, while hardware and software implementation details are described in Section 4. Section 5 presents the results from two separate underwater trials, the first one in a fresh water lake and the second one offshore, in the Atlantic ocean. The paper concludes with a summary and discussion of ongoing work in Section 6.

## 2. Background

Reconstruction of 3D geometry using multiple camera images is a well established area of research [17], popular for a variety of applications. Dense real-time 3D reconstruction is becoming feasible only recently, with the advent of widely available massively parallel commodity general purpose computing on graphics processing units (GPGPU). Notable steps towards real-time 3D reconstruction come from Simultaneous Localisation and Mapping (SLAM) techniques [18], such as MonoSLAM [19] for single camera visual SLAM using sparse features, and the real-time visual odometry system from [20]. Vision-based SLAM has been successfully used underwater, both using monocular [21] and stereo cameras [22].

A different approach from the filter based SLAM systems that preceded it was presented in [23], where the authors separated the camera tracking given a known map from the map update. Following the work of [24], which applied easily parallelisable convex optimisation techniques on commodity GPUs for real-time computer vision applications such as image denoising, Refs. [25,26] presented real-time dense 3D reconstruction pipelines using the feature-based [23] for tracking. The first system to use both dense tracking and mapping and capable of real-time processing was presented by [27].

The work presented in this paper is based on KinectFusion [15,16], a very popular real-time surface reconstruction and camera tracking algorithm designed for RGB + Depth (RGBD) sensors such as the Microsoft Kinect. Although some attempts have been made to use such sensors underwater [28,29], they are extremely limited under such conditions. In order to make the system robust and applicable to existing ROV equipment, this work relies on the application of the KinectFusion algorithm to a stereo camera setup instead of an active sensor, hence the name StereoFusion, as proposed by [30]. Stereo vision has been successfully used underwater for mapping and navigation by various authors [31,32,33].

Alternative methods using acoustic sensors instead of cameras have been explored [34,35]. Most sonars are however not suitable for high precision close range applications due to their low resolution and relatively high minimum operating range. A hybrid vision-acoustic approach has been proposed by [36] using a high-end commercial 3D sonar, which aims to provide the robustness of sonars with the colour information and accuracy of cameras. Such a sonar could be used on its own in a similar way to a depth camera, except for the absence of colour information. As part of an ongoing research project, the implementation of this type of system is currently being trialled with promising results, but its applications are limited due to the previously mentioned limitations of currently available sonars.

## 3. Algorithm

**Figure 1:**
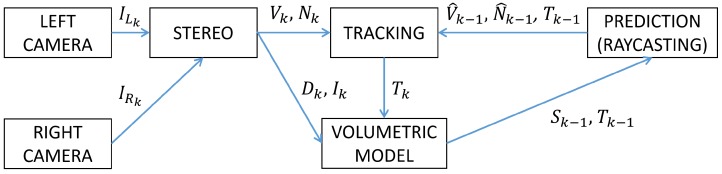
StereoFusion workflow for iteration *k* of the algorithm. Here, $I_{L}$ and $I_{R}$ refer to the stereo pair of RGB images; *D* and *I* are the current depth map and RGB image; *V* and *N* are the vertex map and normal map computed from the depth map; $\hat{V}$ and $\hat{N}$ are the predicted vertex and normal maps; *T* is the camera position; *S* is the Truncated Signed Distance Functions (TSDF).

The base algorithm used in this work is an implementation of KinectFusion [15], a real-time 3D mapping and tracking method developed for use with the Microsoft Kinect and similar RGBD sensors. Although there has been some research done on using range sensors in water [37,38], the results have been of very limited practical use due to difficulties in dealing with light refraction and attenuation, which means that even in good visibility the maximum achievable range is about 20 cm. To overcome this limitation, the work presented in this paper relies on two synchronised colour cameras producing a stereoscopic image pair for disparity estimation. Figure 1 shows the overall workflow of the StereoFusion algorithm.

### 3.1. Stereo

Given a pair of rectified [39] left and right images $I_{L}$ and $I_{R}$, the objective is to find a disparity map [40]$A_{L}$ that provides correspondences for as many pixels as possible:

(1) $$I_{L}\left(u,v\right)\approx I_{R}\left(u+A_{L}\left(u,v\right),v\right).$$

This is achieved using a basic block matching algorithm [40]. For each pixel (u,v), a Sum of Absolute Differences (SAD) is computed between that pixel’s region (the template), and a series of regions in the right image:

(2) $$\mathrm{SAD}\left(u,v,a\right)=\sum_{i=-N_{T}}^{N_{T}}\sum_{j=-N_{T}}^{N_{T}}\left|I_{L}\left(u+i,v+j\right)-I_{R}\left(u+i+a,v+j\right)\right|,$$

where $2N_{T}+1$ is the template size and a∈α⊂R the disparity value for pixel (u,v) in range α. The search is limited to one dimension (*u*) thanks to the epipolar constraint [17]. The pixel’s disparity is then determined by finding the minimum of the SAD:

(3) $$A_{L}\left(u,v\right)=\min_{a}\left\{\mathrm{SAD}(u,v,a)\right\}.$$

This method has been chosen for its computational efficiency. As analysed in [40], the complexity of basic implementations of block matching algorithms is $O(N_{U}N_{A}N_{T})$, where $N_{U}$ is the number of pixels in the image and $N_{A}$ the size of the disparity search range α. By avoiding repeating redundant computations, the complexity can be reduced to $O(N_{U}N_{A})$, thus eliminating the influence of the template’s size.

Once the disparity map $A_{L}$ is known, a range image, or depth map $D_{L}$ can be computed as:

(4) $$D_{L}\left(u,v\right)=f\frac{B}{A_{L}\left(u,v\right)},$$

where *f* is the camera’s focal length and *B* is the baseline, i.e., the spacing between the optical centres of the left and the right camera.

### 3.2. Volumetric Model Representation

The 3D reconstruction is stored as a dense voxel volume, where each voxel contains a Signed Distance Function (SDF) describing its distance to a surface, as described in [41]. The SDF values are positive in front of the surface and negative behind it. Surfaces are therefore extracted from the volume by finding the SDF zero crossings, i.e., the set of points in which the SDF equals zero. In practice, only a truncated version S(p) of the SDF (TSDF) is stored for each point $p\in R^{3}$, such that the true SDF value is only stored within ±μ of the measured value, thus representing the measurement’s uncertainty. The TSDF is represented in each point *p* as:

(5) $$S\left(p\right)=\left[F\left(p\right),W^{F}\left(p\right),C\left(p\right),W^{C}\left(p\right)\right],$$

where F(p) is its value and $W^{F}\left(p\right)$ its weight, the computation of which is described in Equation (7).

Additionally, colour information is stored as C(p) and its corresponding weight $W^{C}\left(p\right)$, in order to be able to make photometric predictions along with the geometric ones.

For a depth map $D_{k}$ with a known pose $T_{k}$, the TSDF at point *p* is computed as:

(6) $$\begin{array}{ccc} F_{D_{k}}\left(p\right) & = & \Psi \left(\lambda^{-1}∥t_{k}-p∥-D_{k}\left(p_{c}\right)\right),\\ \Psi (y) & = & \left\{\begin{array}{cc} \min\left(1,\frac{y}{\mu }\right), & \mathrm{if}\;y\geq -\mu ,\\ \mathrm{null}, & \mathrm{otherwise}, \end{array}\right. \end{array}$$

where $\lambda =∥K^{-1}p_{c}∥$ is a scaling factor for each pixel ray, *K* is the intrinsic matrix, $p_{c}$ is the projection of point *p* onto the camera’s image plane, and $t_{k}$ is the translation vector from $T_{k}$.

For each new depth map $D_{k}$, the global TSDF at point *p* can iteratively be updated as a moving average defined by a threshold $W_{\eta }^{F}$:

(7) $$\begin{array}{ccc} F_{k}\left(p\right) & = & \frac{W_{k-1}^{F}\left(p\right)F_{k-1}\left(p\right)+F_{D_{k}}\left(p\right)}{W_{k-1}^{F}\left(p\right)+1},\\ W_{k}^{F}\left(p\right) & = & \min\left(W_{k-1}^{F}\left(p\right)+1,W_{\eta }\right). \end{array}$$

Compared to a simple running average, this moving average method is robust to dynamic object motion in the scene. The colour TSDF components $C_{k}\left(p\right)$ and $W_{k}^{C}\left(p\right)$ can be updated in the same manner.

### 3.3. Camera Pose Estimation

This section briefly describes the two tracking methods that have been used within the scope of this work.

#### 3.3.1. Depth Tracking

As presented in [15], depth tracking performs camera tracking exclusively based on the depth map from the stereo camera. The newly obtained depth measurements $D_{k}$ are first transformed to a surface measurement composed of a vertex map $V_{k}$ and a normal map $N_{k}$. A surface prediction $\left({\hat{V}}_{k-1},{\hat{N}}_{k-1}\right)$ is generated by raycasting from a viewpoint corresponding to the last known camera position $T_{k-1}$.

An ICP algorithm [42] is used in order to estimate a transformation $T_{k}$, which maps the camera coordinate frame at step *k* to the global frame. It begins by matching points from the surface prediction with the live surface measurement, as detailed in [16]. Given a set of corresponding points, each iteration of the ICP produces a transformation $T_{k}$ minimising a point to plane objective function [43]:

(8) $$\min_{T_{k}}\sum_{p_{c}}∥{\left(T_{k}V_{k}\left(p_{c}\right)-{\hat{V}}_{k-1}\left(p_{c}\right)\right)}^{T}{\hat{N}}_{k-1}\left(p_{c}\right)∥,\;\forall D_{k}\left(p_{c}\right)>0.$$

Assuming small motion between frames, the minimisation is solved according to [44].

#### 3.3.2. Colour Tracking

The colour tracker, as described in [45], relies on the live RGB image $I_{k}$, rather than on the depth map $D_{k}$. The first step of the tracker consists of creating a model based prediction of surface points ${\hat{V}}_{k-1}$ and their corresponding colour values ${\hat{C}}_{k-1}$. The prediction is again done from a viewpoint corresponding to the camera position at step k−1, like for the depth tracker described in Section 3.3.1. The cost function to be minimised in this case, however, is an $\ell^{2}$ norm of the difference between the colour of a predicted point and the colour of the corresponding pixel in $I_{k}$:

(9) $$\min_{T_{k}}\sum_{p_{c}}∥I_{k}\left(KT_{k}{\hat{V}}_{k-1}\left(p_{c}\right)\right)-{\hat{C}}_{k-1}\left(p_{c}\right)∥.$$

The minimisation is solved using the Levenberg–Marquardt algorithm [46].

### 3.4. Raycasting

Raycasting [47] is used to obtain surface predictions both for camera tracking, as seen in Section 3.3, and for visualisation of the model. The process renders the zero level set $F_{k}\left(p\right)=0$ of the current TSDF into a viewpoint with position $T_{r}$.

A virtual ray $T_{r}K^{-1}p_{c}$ is generated for each pixel $p_{c}$ of the image being rendered. The algorithm steps through the volume along each ray, looking for a change in the sign of the TSDF values. If the TSDF values have changed from positive to negative, a surface interface has successfully been detected, which provides the data for the pixel being rendered.

## 4. Implementation

### 4.1. Software

The software implementation is based on the InfiniTAM [48] framework, adapted to work with the Robot Operating System (ROS) [49] and a stereo camera as the input device. All steps of the KinectFusion algorithm are very well suited for parallel execution, as described in [16]. For this reason, all computation is performed on a Graphics Processing Unit (GPU). Modern consumer grade GPUs typically consist of several hundreds or thousands of processing units. From a general purpose programming point of view, GPUs can be considered SIMD (single instruction, multiple data) devices. This means that optimal performance is achieved in cases when the same computation needs to be performed on a large number of inputs. Every StereoFusion step can be parallelised in such manner, performing the same operation either for each image pixel or for each voxel in the model volume, making it perfectly suited for this type of hardware. For the purpose of this work, everything has been implemented using Nvidia CUDA. The test GPU is an Nvidia GTX 980 Ti, which has 2816 CUDA cores and 6 GB of global memory. Other relevant components of the computer used are an Intel i7-4930k processor and 16 GB of RAM. The software has been running on Ubuntu 16.04 with ROS Kinetic Kame.

### 4.2. Stereo Rig

**Figure 2:**
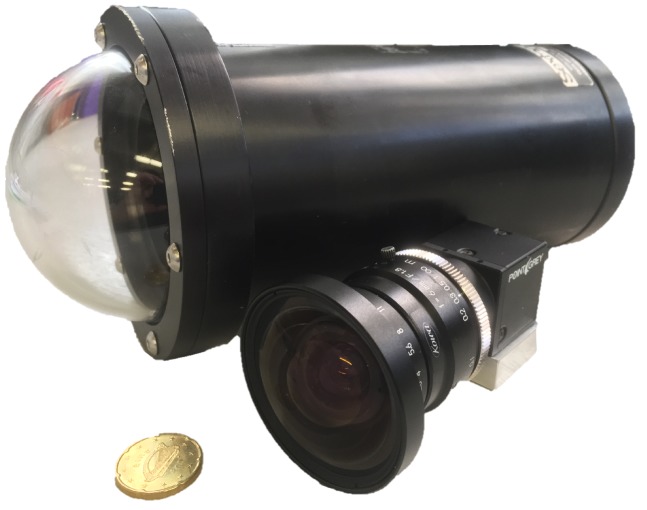
Camera and underwater housing.

**Figure 3:**
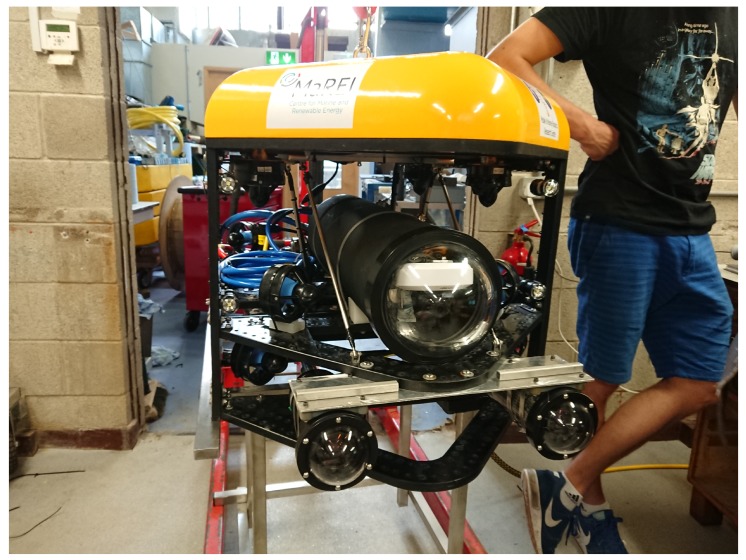
University of Limerick ROV Áed with the stereo camera setup mounted below the main piloting camera.

**Figure 4:**
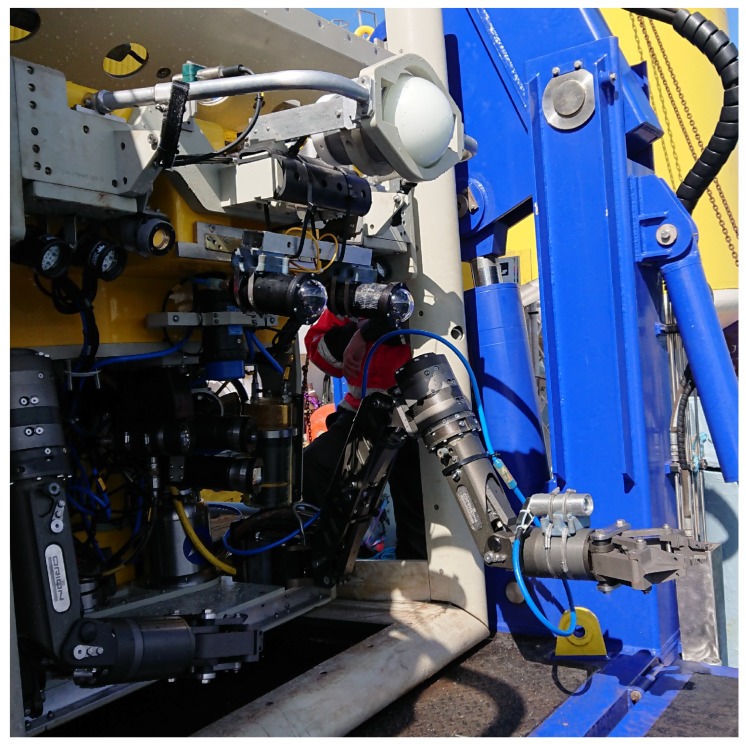
University of Limerick ROV Étaín with the stereo camera setup mounted above the manipulators.

The algorithm described in Section 3 has been tested using a stereo RGB camera rig. It consists of a pair of FLIR (formerly Point Grey) Blackfly GigE Vision cameras (BFLY-PGE-23S6C-C), with global shutter Sony IMX249 1/1.2” CMOS sensors. These are standard industrial machine vision cameras enclosed in underwater housings rated to a water depth of 1000 m. The camera and the housing can be seen in Figure 2. The stereo rig was mounted on the front of two ROVs, as shown in Figure 3 and Figure 4. The shutters are synchronised through the cameras’ GPIO (General-Purpose Input/Output) pins as described in [50], where one camera emits a signal that triggers the second one, without the need of an external signal generator. This is necessary in order to accurately estimate disparities from a stereo image when motion is involved.

All the processing is performed on the surface, using a dedicated computer, described in Section 4.1, located in the ROV control cabin. In order to communicate with the surface PC through the two kilometres long ROV umbilical, the Gigabit Ethernet connections are transformed from copper to optical fibre connections and back to copper using TP-Link Gigabit SFP Media Converters. Both cameras rely on Power over Ethernet (PoE), which is injected to the Gigabit Ethernet lines on-board the ROV.

Two Kowa LM6HC wide angle lenses are used in order to have the widest possible field of view (FOV). Despite the lenses, the FOV would be reduced due to light refraction if the cameras were behind flat ports, for this reason both enclosures use dome ports. Having a wide FOV allows a relatively large stereo baseline (40 cm in this case) without sacrificing close range measurements. It is also important for guaranteeing overlap between consecutive images, and aiding the vision-based tracking algorithm by reducing the probability of completely losing sight of the observed scene.

## 5. Results

The algorithm discussed in Section 3 has been tested on two ROV systems under different conditions. In both cases, the cameras produced RGB images with a resolution of 960×600 pixels at 22 frames per second. The frames are processed in real time and for all the presented results the 3D models are being built and updated on-line. The voxel size used for all the presented models is 5 millimetres.

### 5.1. Good Visibility, Fresh Water

The main results presented in this work have been obtained during trials with an inspection class ROV in a flooded quarry near Portroe, County Tipperary, Ireland. This quarry is normally used as a scuba diving centre. Various items have been placed underwater for the entertainment of divers (e.g., a van, a boat, a car, a bar, computers, etc.). These are interesting targets to test the described algorithms, thanks to their complex geometry and texture. Additionally, using familiar targets facilitates qualitative analysis of the 3D models.

The vehicle used, called ROV Áed and shown in Figure 3, is a custom system built in-house at the Centre for Robotics and Intelligent Systems (CRIS) at the University of Limerick (UL). It is intended to be a lightweight highly manoeuvrable inspection ROV capable of operating in strong currents and other challenging environments.

**Figure 5:**
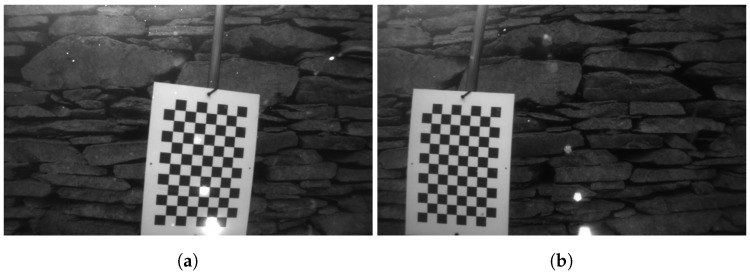
Example of a stereo pair used for camera calibration. (**a**) left camera; (**b**) right camera.

The cameras have been calibrated underwater on-site, using a 7 × 11 chessboard with the sides of each square measuring 280 mm, as shown in Figure 5. The chessboard has been printed on an A3 sized (297×420 mm) PVC board, which was then attached on a pole in order to submerge it and move it manually from the side of a pier. The cameras have been calibrated using a pinhole camera model, and the distortion corrected using the plumb bob model (radial polynomial + thin prism model) [51].

**Figure 6:**
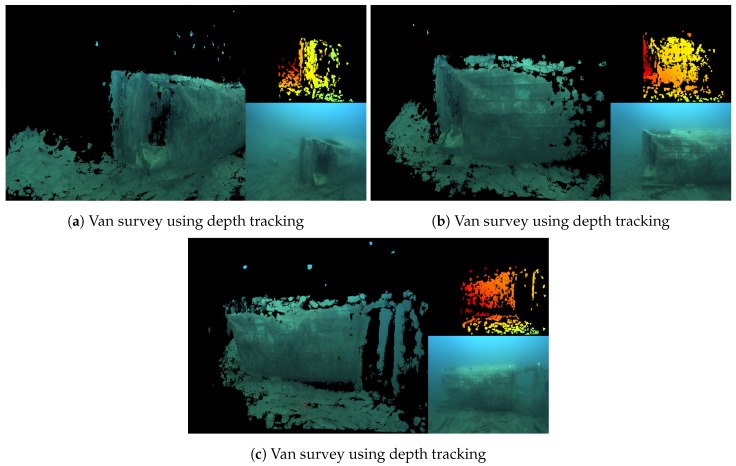
Van reconstruction in good visibility. The main panel shows the 3D model, the top right shows the depth maps, and the bottom right shows the original colour image from the left camera.

Figure 6 shows a series of images from a survey of a submerged van, about 4 m in length. It displays the 3D model, the current frame from the left camera of the stereo pair, and the depth map calculated in the left camera’s reference frame. The model has been built incrementally, as the ROV was manually piloted around the van. The camera position was continuously estimated using the depth tracking method described in Section 3.3.1.

**Figure 7:**
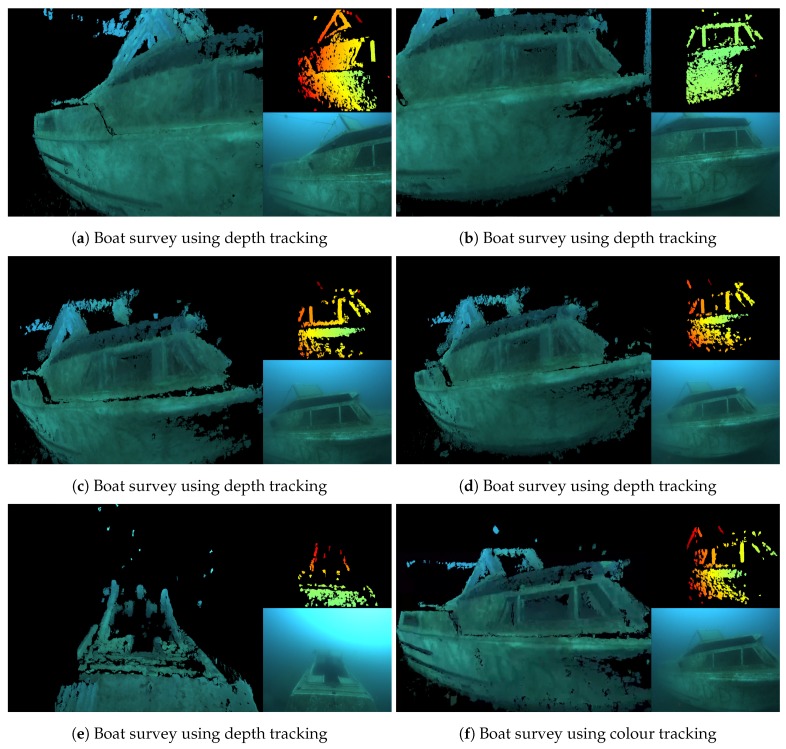
Boat reconstruction in good visibility.

Figure 7a–e show images of the algorithm running during a survey of a submerged boat, about 6 m in length. Figure 7f is from a different survey of the same boat, but this time using the colour tracker from Section 3.3.2. In the quarry, both trackers performed equally well, producing good reconstructions and providing reliable camera tracking.

### 5.2. Bad Visibility, Sea Water

**Figure 8:**
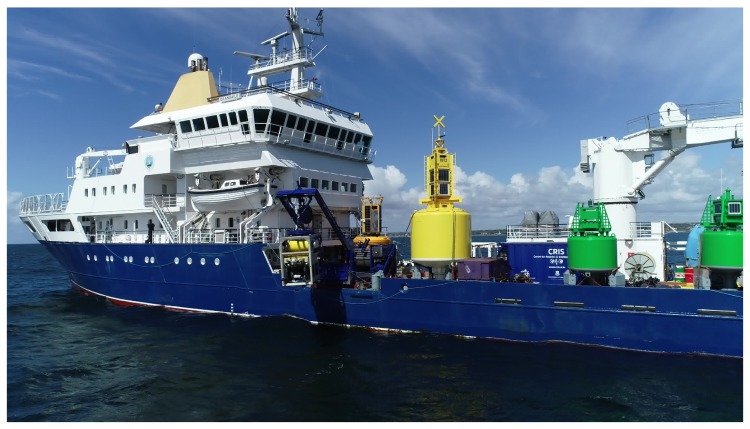
ROV Étaín inside its Tether Management System, being deployed from the ILV Granuaile.

**Figure 9:**
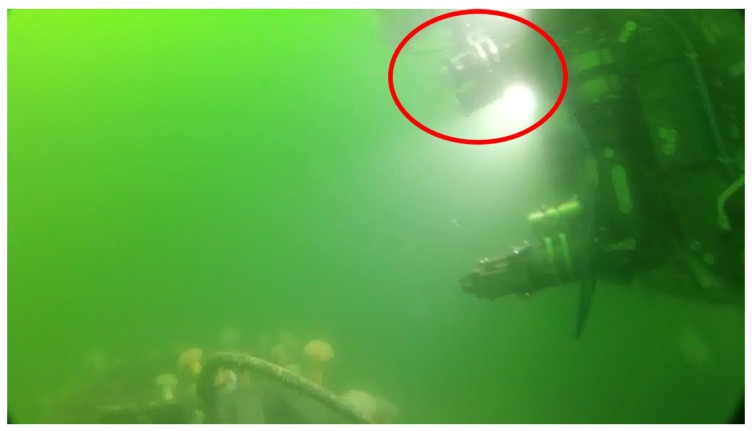
The stereo rig (red) on the ROV in bad visibility conditions.

Testing has been carried out at sea in Galway Bay, Ireland, with the stereo rig mounted on the UL work class ROV Étaín. This ROV is a commercial Sub-Atlantic Comanche system, it was launched from the Irish Lights Vessel (ILV) Granuaile, shown in Figure 8. The target surveyed in this scenario is a 2 metre tall metal frame with panels that simulate valves, which is used to test manipulator control algorithms. Visibility in this case was low, with a lot of particles being moved around by the strong tidal current, as can be seen in Figure 9.

**Figure 10:**
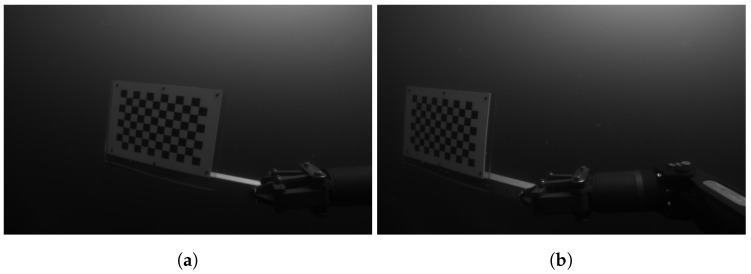
Example of a stereo pair used for camera calibration with the manipulator. (**a**) left camera; (**b**) right camera.

The stereo cameras have been calibrated underwater using the same chessboard and models as described in Section 5.1. In this case, however, due to physical constraints and safety considerations, the chessboard could not be moved manually from the side of the ship, therefore it had to be operated by one of the two Schilling Orion 7P hydraulic manipulators that are on board the ROV, as shown in Figure 10. Obtaining the necessary images, this way proved to be very challenging and time consuming for the pilot, mostly due to the manipulator’s inaccurate control and limited motion capabilities. A consideration for future operations would be to automate the calibration procedure by predefining the manipulator’s trajectory using the Cartesian control presented in [10].

**Figure 11:**
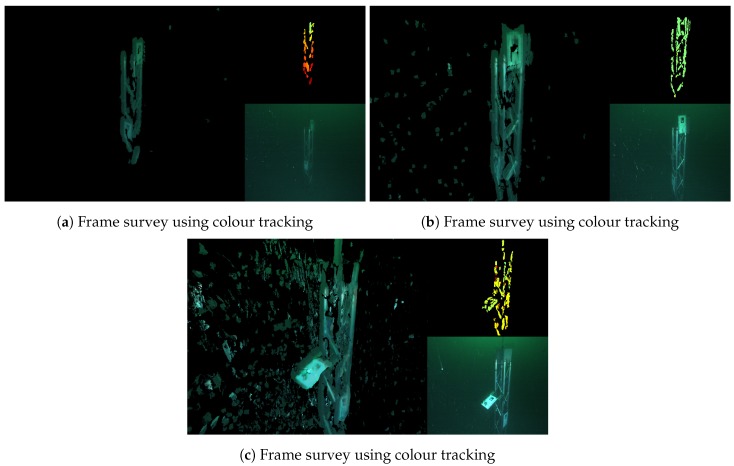
Metal frame reconstruction in bad visibility. The main panels show the 3D model, the top right show the range images, and the bottom right show the original colour image from the left camera. (**a**) approaching the target; (**b**) target approached; (**c**) after moving $90^{{}^{\circ}}$ clockwise around the target.

Figure 11 shows snapshots of the survey, which was performed by manually piloting the ROV in a circle around the target. Unlike in the scenario described in Section 5.1, in this case, the colour tracker proved to have a significant advantage over the depth tracker. This is due to the target’s geometry, i.e., the beams from which the metal frame is composed are relatively thin and do not always appear clearly in the depth map.

The effect of the floating particles is visible in the reconstruction, especially in Figure 11c, which is filled with tiny artefacts. Apart from the effects on the 3D model itself, this poses a serious challenge for camera tracking, which in this case was pushed to its limits. Due to frequent tracking failure, the survey had to be performed several times before a full circle around the target was successfully accomplished. Although a model has been obtained, it is clear that a purely vision based system is not robust enough for reliable operation under such challenging conditions.

### 5.3. Qualitative Comparison to Post-Processed Photogrammetry

**Figure 12:**
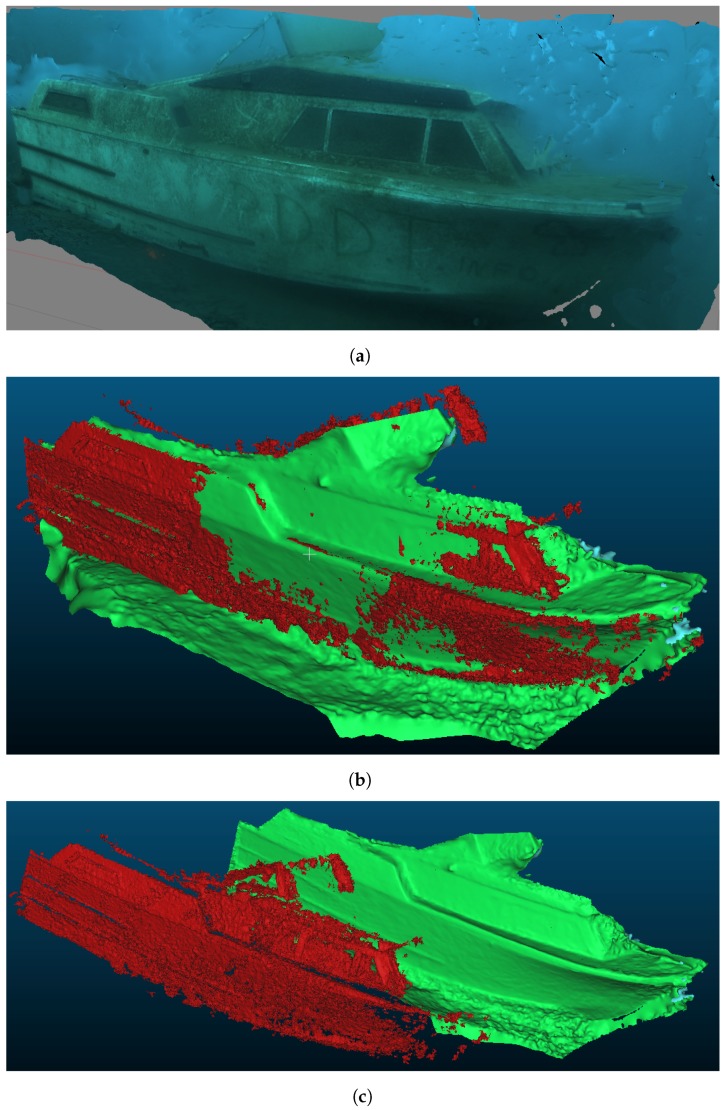
Qualitative comparison between StereoFusion and post-processed photogrammetry. The software used for photogrammetry is Agisoft PhotoScan. (**a**) textured model obtained from the boat sequence using Agisoft PhotoScan; (**b**) overlapping models: the green one is built using Agisoft PhotoScan and the red one using StereoFusion; (**c**) models side-by-side: the green one is built using Agisoft PhotoScan and the red one using StereoFusion.

Although StereoFusion does not aim to challenge post-processing techniques in terms of reconstruction quality, but rather produce usable models in real time, a comparison is nonetheless useful in order to estimate its results. Figure 12 shows a qualitative comparison of the 3D model of the boat from Figure 7 with one obtained using the same image sequence with a post-processing photogrammetry technique. The post-processed model, displayed in Figure 12a, has been generated using Agisoft PhotoScan [52], a widely used commercial photogrammetry tool. Figure 12b presents a comparison of the two models aligned with each other, where the green one is the result of post-processing while the red one is produced in real-time by StereoFusion. For better understanding, both models are shown in Figure 12c side-by-side. From this comparison, it is clear that the geometry produced by StereoFusion matches the one obtained in post-processing, although not perfectly. It is important to note that the post-processed model is only used for qualitative comparison, as it does not represent ground truth. The exact geometry of the sunken boat used in this sequence is unknown. However, the targets presented in this work have been intentionally chosen because they are familiar objects, thus making a qualitative analysis of the presented results more intuitive to the reader.

From these comparative results, StereoFusion can be considered applicable to the underwater domain. The presented real-time data could be useful in applications such as obstacle avoidance, path planning, target referenced localisation, object detection, and augmented reality for enhancing the pilot’s perception.

## 6. Conclusions and Future Work

This paper presented underwater StereoFusion, an adaptation of KinectFusion which instead of a Kinect uses a stereo camera. The software has been tested in two underwater scenarios, using different vehicles. In good visibility, the system proved capable of reliable operation at video frame rate. Because it provides real-time dense 3D reconstruction of targets and relative camera tracking, it opens a variety of new possibilities in underwater robotics. Such a system can be used for applications such as online verification of survey data, navigation relative to a target, semi and fully autonomous manipulation, path planning, obstacle avoidance, etc.

As with most underwater vision systems, its main problem is operation in low visibility environments, as shown in Section 5.2. Although still capable of producing a 3D model, the quality of both the reconstruction and the tracking significantly decreases. One way to tackle this will be to aid the camera tracking by exploiting navigation data from the onboard inertial navigation system. Fusing inertial with vision-based navigation will improve both the estimation quality and the system’s robustness. Additionally, the system can be made more robust to low visibility by using a 3D sonar, either instead of the stereo camera, or by fusing the acoustic with vision data in a manner similar to that described by [36]. Ongoing testing will provide information on the quality of a 3D sonar used as the only sensor, using the same algorithm described in this paper.

Further ongoing development of the vision-based system is focused on a monocular implementation, relying on the work of [27,53]. This is particularly relevant for underwater manipulation automation for multiple reasons. Existing manipulators in the global work class ROV fleet are typically equipped with a single camera mounted near the gripper (Figure 4), and are not suited to host a stereo rig due to its size. In addition to mechanical considerations, a fixed baseline stereo system is not well suited for operation at significantly different distances from the target. As discussed by the authors in [9], in order to automate manipulation tasks, it is necessary to reconstruct the scene from a distance while also being able to continuously keep track of the camera as the manipulator approaches its target. These limitations of stereo methods can be solved by monocular systems.

## Abbreviations

The following abbreviations are used in this manuscript:

ROV	Remotely operated vehicle
AUV	Autonomous underwater vehicle
SLAM	Simultaneous localisation and mapping
GPU	Graphics processing unit
GPGPU	General purpose GPU
ICP	Iterative closest point
SDF	Signed distance function
TSDF	Truncated SDF
FOV	Field of view
RGB	Red, green, blue
RGBD	RGB + depth
CMOS	Complementary Metal-Oxide-Semiconductor
